# An integrated microfluidic platform for nucleic acid testing

**DOI:** 10.1038/s41378-024-00677-6

**Published:** 2024-05-23

**Authors:** Antao Sun, Petra Vopařilová, Xiaocheng Liu, Bingqian Kou, Tomáš Řezníček, Tomáš Lednický, Sheng Ni, Jiří Kudr, Ondřej Zítka, Zdenka Fohlerová, Petr Pajer, Haoqing Zhang, Pavel Neužil

**Affiliations:** 1 Ministry of Education Key Laboratory of Micro and Nano Systems for Aerospace; School of Mechanical Engineering, Northwestern Polytechnical University, 127 West Youyi Road, Xi’an, Shaanxi 710072 P. R. China; 2 Department of Chemistry and Biochemistry, Mendel University in Brno, Zemědělská 1, 61300 Brno, Czech Republic; 3 ITD Tech s.r.o, Osvoboditelů 1005, 735 81 Bohumín, Czech Republic; 4 Central European Institute of Technology, Brno University of Technology, Purkyňova 123, Brno, 61200 Czech Republic; 5 Department of Electronic and Computer Engineering, The Hong Kong University of Science and Technology, Clear Water Bay, Kowloon, Hong Kong SAR, China; 6 Department of Microelectronics, Faculty of Electrical Engineering and Communication, Brno University of Technology, Technická 3058/10, Brno, 61600 Czech Republic; 7 Military Health Institute, U Vojenské nemocnice 1200, 16200 Praha 6, Czech Republic; 8 The Key Laboratory of Biomedical Information Engineering of Ministry of Education, School of Life Science and Technology, Xi’an Jiaotong University, Xi’an, Shaanxi 710049 P. R. China; 9 Bioinspired Engineering and Biomechanics Center (BEBC), Xi’an Jiaotong University, Xi’an, 710049 P. R. China

**Keywords:** Chemistry, Electrical and electronic engineering, Microfluidics

## Abstract

This study presents a rapid and versatile low-cost sample-to-answer system for SARS-CoV-2 diagnostics. The system integrates the extraction and purification of nucleic acids, followed by amplification via either reverse transcription-quantitative polymerase chain reaction (RT–qPCR) or reverse transcription loop-mediated isothermal amplification (RT–LAMP). By meeting diverse diagnostic and reagent needs, the platform yields testing results that closely align with those of commercial RT-LAMP and RT‒qPCR systems. Notable advantages of our system include its speed and cost-effectiveness. The assay is completed within 28 min, including sample loading (5 min), ribonucleic acid (RNA) extraction (3 min), and RT-LAMP (20 min). The cost of each assay is ≈ $9.5, and this pricing is competitive against that of Food and Drug Administration (FDA)-approved commercial alternatives. Although some RNA loss during on-chip extraction is observed, the platform maintains a potential limit of detection lower than 297 copies. Portability makes the system particularly useful in environments where centralized laboratories are either unavailable or inconveniently located. Another key feature is the platform’s versatility, allowing users to choose between RT‒qPCR or RT‒LAMP tests based on specific requirements.

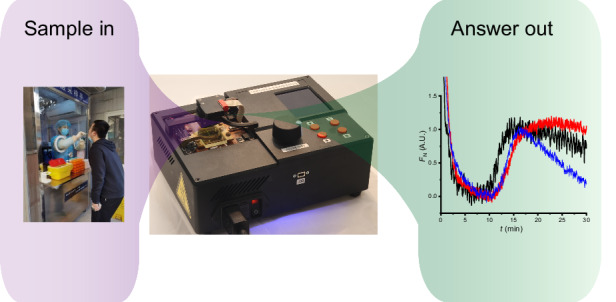

## Introduction

The outbreak of coronavirus disease 2019 (COVID-19), which resulted from severe acute respiratory syndrome coronavirus 2 (SARS-CoV-2) infection at the end of 2019, posed significant challenges to medical systems worldwide. Societal vulnerability in the face of a pandemic was highlighted by this event. In most countries, access to large-scale detection of COVID-19 and subsequent treatment remains limited. Countries that developed such capabilities experienced a massive shift in resource allocation and significant disruptions to daily life^1^. The pandemic has reinforced the importance of preventive measures, specifically the need for continuous pathogen surveillance and prompt quarantine measures, to curb the transmission of infectious diseases.

Numerous methods are available for virus detection^2^, and the most commonly used methods are antibody detection^3^ and nucleic acid amplification tests (NAATs)^4^. Once invaded by SARS-CoV-2, the human body produces IgM or IgG antibodies for defense. Methods for antibody detection measure the levels of these antibodies in blood samples, providing an indirect indication of the presence of SARS-CoV-2. This method of detection, which does not require cell culture, can be completed within minutes. However, the production of detectable antibody levels may not occur in the early stages of infection, complicating the confirmation of SARS-CoV-2 infection. In contrast, NAATs can label and amplify viral sequences with fluorescent or other markers in vitro, facilitating the detection of the virus regardless of the infection stage. Thus, NAATs prove to be relatively suitable for detecting SARS-CoV-2, especially in early infection stages.

Viral diagnostics typically involve the extraction of ribonucleic acid (RNA) and its subsequent detection. Initially, RNA is isolated and purified from a nasal or throat swab. This process is followed by either reverse transcription-quantitative polymerase chain reaction (RT–qPCR) or reverse transcription loop-mediated isothermal amplification (RT–LAMP), techniques that amplify RNA to a fluorescently detectable level. In recent years, developed instruments, both commercial and prototype instruments, have combined RNA extraction and detection via either RT‒PCR or RT‒LAMP to obtain a sample-to-answer diagnosis^5^. Such instruments reduce the potential for cross-contamination during sample transfer between instruments and enhance diagnostic efficiency^6^. The market has offered quite a few sample-to-answer systems for COVID-19 diagnostics, including the Vivalytic Analyser (Bosch GmbH, Germany)^7^, Accula™ (Mesa Biotech, Inc., San Diego, CA, U.S.A.)^8^, and Cobas® Liat® (Roche Diagnostics International AG, Basel, Switzerland) instrument^9^. These systems perform the required tests as well as scan test cartridge identification codes, linking test results with patient data to expedite the diagnostic process^10–12^. The Convergys® RT‒PCR COVID‒19 testing platform can be used to conduct a fluorescence test within 90 min^13^. A system utilizing RT-LAMP has been shown to be capable of detecting as few as 10 to 100 RNA copies within one hour^14^, automating the process through the use of magnetic beads for nucleic acid extraction. However, the size and complexity of these instruments, along with their cost, make them impractical for point-of-care testing (POCT) and challenging to use in outdoor conditions. Furthermore, the demanding operation and long detection times of POCT systems have hindered their broader adoption.

Microfluidic techniques are well suited for POCT applications due to their simple structure, compact size, and affordability. Integrated off-center microfluidic platforms can store and release reagents, whereas most centrifugal systems depend on valves^15,16^, resulting in complexity and higher manufacturing costs^17–23^. Rapid paper-based diagnostic systems exist for DNA capture through electrostatic adsorption onto filter paper fibers, but these often exhibit less than 80% efficiency^24–26^. Microarray-based DNA detection systems capable of simultaneously diagnosing multiple viruses are also available. However, their long production cycles and high costs make them less ideal for POC diagnostics^27–31^. Recent developments in sample-to-answer systems for COVID-19 diagnosis include rapid testing, high-throughput methods, and automated detection^32–34^. However, these systems have not become commonplace due to their cost, size, or design deficiencies. Thus, simple, rapid and low-cost diagnosis remains a significant challenge for the application of microfluidic sample-to-answer systems.

**Fig. 1:**
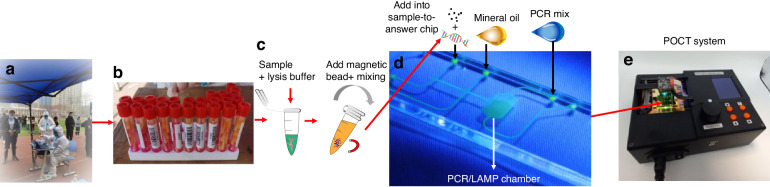
Workflow of a microfluidic diagnostic system for RNA detection from sample collection to result display. **a** Sample collection: A sample is collected under an outdoor tent. **b** Reagent setup: Test tubes filled with various reagents are used for RNA detection. **c** RNA preparation protocol: A flow diagram details the steps of the RNA preparation process using magnetic beads. **d** Sample extraction and subsequent amplification on a microfluidic chip: An illustration of the microfluidic chip shows the sample and reagents loaded into the corresponding port. **e** Photograph of the POCT microfluidic system: A vertical view of the microfluidic diagnostic device, highlighting its overall features with a chip in place for analysis

Here, we developed a rapid, versatile, and low-cost sample-to-answer system for SARS-CoV-2 diagnostics. The system consists of RNA extraction using magnetic beads, reverse transcription, and subsequent amplification and detection. Compared with commercial sample-to-answer systems for COVID-19 diagnostics approved by the Food and Drug Administration (FDA), the proposed system can implement both PCR and LAMP methods based on diagnostic and reagent requirements (Fig. 1). The major advantage of LAMP is its ability to function with minimal power consumption. Designing hardware specifically for LAMP requires thermal insulation of the heated sections. Running PCR on such a system would result in a markedly slower process. Consequently, using a real-time PCR system to run LAMP has limited practicality, as it utilizes the advantages of the simple system of LAMP compared to that of PCR. However, demonstrating the system’s ability to perform both PCR and LAMP analyses is crucial, highlighting its versatility for POCT. The distinctive features of LAMP include rapid amplification speed relative to traditional PCR and straightforward end-point detection methods such as turbidity^35^ or colorimetry^36^. In contrast, PCR offers the flexibility to design multiplex assays within a single reaction.

Additionally, our system can complete the entire assay within 28 min, including sample loading (5 min), RNA extraction (3 min), and RT-LAMP (20 min), making it competitive with most FDA-approved devices (Table 1). The poly(methyl methacrylate) (PMMA)-based sample-to-answer chip allows for mass production without complex treatment procedures, significantly reducing the cost of a single test to ≈$9.5, which is much less than that of commercial alternatives. Further cost reductions are achievable through mass production techniques such as roll-to-roll hot embossing^37^ or injection molding^38^. A key advantage of our compact, portable system is its ability to provide immediate diagnoses at the point of interest, eliminating the need to transport samples to a centralized facility.

**Table 1:** Comparison of sample-to-answer systems

Name	Price	Method	Time	LoD	Advantages	Disadvantages
Lucira COVID-19 & Flu Test	$75 (kit)	RT-LAMP	30 min	not listed	98% accuracy, early detection	18 months shelf life
Metrix COVID-19 Test	Not listed	RT-LAMP	30 min	667 copies·mL^−1^	portable device	only for SARS-CoV-2
UOL COVID-19 Molecular Test	$540 (kit), $249 (instrument)	RT-LAMP	12–40 min	400 copies per swab	portable device	risk of cross contamination
Cobas® Liat System	$9900 (instrument)	RT‒PCR	20 min	12 copies·mL^−1^	fast, sensitive	risk of the assay tubes leakage, abnormal PCR amplification curve^63^
BD MAX™ System	$221,283 (instrument)	PCR	<3 h	not listed	fully integrated automated system	enormous size and expensive
Proposed system in this paper	$9.5 single test	RT‒PCR & RT-LAMP	28 min	not listed	fast, cost-effective	

## Materials and methods

### System setup

**Fig. 2:**
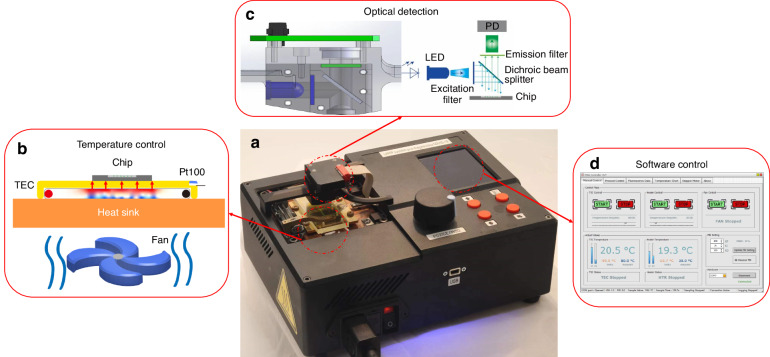
Components of the microfluidic diagnostic system. **a** System overview: The microfluidic diagnostic device featuring a stepper motor assembly and user interface with operational buttons and display. **b** Cross-sectional view of the temperature system: Illustration depicting the temperature system containing a thermoelectric cooler (TEC), Pt100 temperature sensor and heat sink with an electric fan. **c** Cross-sectional view of the optical system (left) and optical pathway schematic (right): diagram showing the LED light source passing through an excitation filter to the sample, with the emitted light collected by a photodiode (PD) after passing through an emission filter. **d** Software control user interface

The system comprised a sample-to-answer chip, a temperature control module, and an optical detection module (Fig. 2). A single-chip controller with built-in firmware controls all system operations, including magnet motion, temperature control, and fluorescence detection. Users can program all steps using four buttons at the POCT unit, and a rotating wheel allows for manual control of magnet locations. For added convenience, the system included a universal serial bus (USB) interface to facilitate communication with a personal computer (PC). A block diagram is available in Supplementary Section A.

### Sample-to-answer chip

#### Chip design and fabrication

**Fig. 3:**
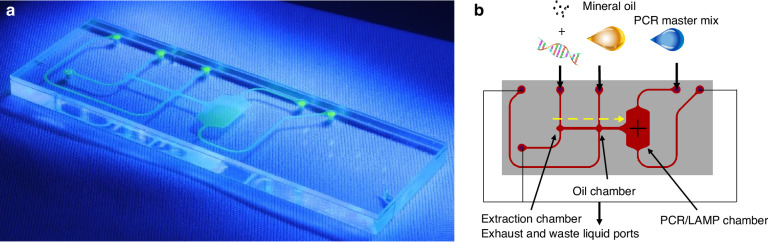
Microfluidic chip for RNA detection and schematic overview. **a** Actual chip: A photograph of the microfluidic chip filled with fluorescein illuminated with blue light, thus increasing the contrast of all microfluidics channels. **b** Schematic diagram: The diagram illustrates the microfluidic pathway within the chip, detailing the flow (yellow dashed line) of RNA samples through various processing stages, including extraction, oil, and PCR/LAMP chambers

We used a JAVA-based script in Nanolithography Toolbox software^39^ with integrated microfluidics features^40^ to design the chip, generating a file in the graphic design system II (GDS II) format. The design included features tailored for microfluidics, such as a chamber with tangential connections and a rounded path for fluid delivery, ensuring minimal dead volume^22^. The PCR/LAMP chamber, the system’s main feature, had a nominal volume of 6 μl and an assumed depth of 100 μm. Additionally, the system included two smaller chambers: one for capturing magnetic particles and the other for purifying these particles with mineral oil to remove debris produced by cell/virus lysis (Fig. 3). We transformed the GDS II file into a data exchange format and then into a product model data format, commonly used for computer numerical control (CNC) machining. A ≈ 3 mm-thick PMMA chip with an area of 25 × 75 mm², was vertically milled to form the microfluidic channels. The vertical and horizontal channels had widths of ≈955 µm and ≈1350 µm, respectively. This CNC-fabricated chip was then bonded to a ≈ 1 mm thick flat piece of PMMA through chemically assisted thermal bonding following the protocol described in 2007 ^41^.

#### Sample in-chip workflow

We used an adsorption technique with magnetic nanoparticles for RNA extraction, which involved several key steps: virus lysis, RNA/DNA adsorption, debris washing, and RNA/DNA elution. Off-chip, we spiked synthetic viral RNA into lysis buffer containing magnetic nanoparticles^42^. In this step, RNA was released from the lysed cells and adsorbed onto the magnetic nanoparticle surface. The RNA-magnetic nanoparticle (RNA-MNP) complex was then sequentially pumped into the chip, where a permanent magnet mounted on an arm attached to a stepper motor in the system trapped and moved the RNA-MNPs. In the second step, we washed the RNA-MNP complex in the oil chamber using M5904 mineral oil (Sigma‒Aldrich, MO, U.S.A.) to remove PCR/LAMP inhibitors attached to the magnetic nanoparticles. Subsequently, we moved the complex to the final position—the PCR/LAMP chamber—where the RNA template was mixed with the master mix. We also used the same oil to block all the microfluidic chip input ports to prevent sample evaporation and removal of the chamber during amplification. Isothermal reverse transcription (RT) of RNA and complementary DNA amplification were performed by RT-LAMP, and we detected the resulting amplicons using a fluorescence-based method.

### Temperature control

We heated/cooled the microfluidic chip using a thermoelectric cooler (TEC) powered by an H-bridge with pulse-width modulation (PWM) that used electrical current pulses at a nominal frequency of 100 kHz. We controlled the dissipated power through the duty cycle modulation of the PWM. The direction of the electrical current determines whether the TEC is cooling or heating. We equipped the top plate of the TEC with a Pt100-type temperature sensor integrated into a closed feedback loop system for proportional integrative derivative (PID) control.

### Optical detection

We developed and tested a single-point fluorescent detection system using a light-emitting diode (LED) with a nominal wavelength of 465 nm and a luminous intensity of 4.7 cd (Fig. 2). This system was powered by electrical current pulses set to an amplitude of 200 mA, created by 5 V pulses and a series resistor with a nominal value of 10 Ω. The pulse frequency was set to 998 Hz, with a duty cycle of 10%. The optical system included a fluorescein isothiocyanate (FITC) filter set comprising three filters. The low-pass filter, with a nominal cutoff wavelength of 490 nm, prevented the passage of light with a longer wavelength. Subsequently, a longpass filter with a cutoff wavelength of 500 nm served as a dichroic mirror, reflecting the light. We then focused the light into the PCR/LAMP chamber of the microfluidic chip through a lens with a 6.35-mm diameter, a 1.8-mm focal length, a 1.8-mm working distance, and a 0.7-mm numerical aperture.

The light from the excited fluorescence passed through a dichroic mirror and was then filtered through a bandpass filter with a nominal bandwidth of (525 ± 25) nm before being detected by a photodiode (PD) detector. We first converted the generated photocurrent into voltage using a transimpedance amplifier with a conversion factor of 3.3 × 10^8^. A lock-in amplifier then processes the output signal. We used a computer-aided design system to design the optical system hardware. Both the system casing and optical housing were fabricated using a CNC system and subsequently assembled, as detailed in Supplementary Section B.

Our control software enabled the adjustment of the lock-in amplifier’s parameters, such as background removal (compensation) and phase shift, to optimize the system’s gain. We determined that the optimal phase shift for the system was 73°, with the background removal setting adjusted to 920 (Supplementary Section B).

### Software control

Upon connecting the system to a PC through a USB interface and initiating the software, several windows became available. These included the *Welcome* window, *Manual Control*, *Protocol Control*, *Fluorescence Data*, *Temperature Chart*, and *Stepper Motor Control* windows, as detailed in Supplementary Section C.

When the program was initiated, users first encountered the *Welcome* window, which led them to the *Manual Control* page. Here, the software established a connection with the hardware using the USB communication interface. Once the connection was successful, indicated by the connection icon turning green, the program displayed the last PID values from the sample-to-answer system. Users had the discretion to modify the PID constants if needed. The program also allowed manual setting of the TEC and the TEC support temperatures, along with activation of the cooling fan.

The *Protocol Control* window, designed to be self-explanatory, allowed users to select data for the RT‒PCR protocol. Users can hasten the system by incorporating preannealing and preelongation steps. Users also had the option to use the RT-LAMP protocol, keeping the temperature constant during both the denaturation and annealing/elongation phases. Once a protocol was defined, it could be uploaded to the sample-to-answer system. Alternatively, the protocol can be stored on a PC and transferred to or from the sample-to-answer system as needed. The PC screen showed the selected temperature, actual TEC temperature, temperature difference, step name, remaining time for the step, and number of cycles. An icon was also presented to commence and stop the protocol.

In the *Fluorescence Data* window, users can fine-tune the lock-in amplifier parameters to compensate for background noise and adjust the phase shift for optimal gain. The system also displayed the actual fluorescence analog-to-digital conversion value at the lock-in amplifier output and recorded it in a file.

The *Temperature Chart* window allowed users to control the temperature recording frequency and adjust the sampling rate between 0.1 s and 1.0 s. Users can read the temperatures of both the TEC plate, where the microfluidic chip was situated, and the substrate. Since increasing the temperature of the bottom part of the TEC enhances the heating rate and reduces the cooling rate, users can optimize the system by using substrate temperature control for the quickest heating/cooling rate. The data can be stored in a text file on the PC.

The *Stepper Control* window enables users to manually maneuver the magnet using the large knob in the sample-to-answer system or to control it semiautomatically for hands-free detection of RNA. This window displays the stepper motor location and status and allows users to set the stepper motor parameters. When initiating the motor, we set the arm to move from a step position of 1000 to 9500; subsequently, we used the wheel to control the motor position. Alternatively, the motor can be controlled by software as well to achieve fully automated operation.

### Experimental protocol

#### Sample Preparation and Nucleic Acid Purification

For all the experiments, we utilized the quantitative synthetic N gene of SARS-CoV-2 RNA supplied by Genewiz, Inc. (Suzhou, China), with a stock solution concentration of 3.46 × 10^12^ copies·µl^−1^. We extracted viral RNA using SeraSil-Mag 400 (Cytiva, MA, U.S.A.) superparamagnetic coated silica beads. We spiked 100 µl of RNA into 200 µl of RNeasy Lysis Buffer (buffer RLT) from the RNeasy Mini Kit (Qiagen, catalog number: 74004, Hilden, Germany) and added 10 µl of magnetic nanoparticles at a concentration of 20 mg·µl^−1^ to the water. After manually mixing the lysate, we pumped the sample into the microfluidic chip. We then washed the RNA-MNP complex with 10 µl of M5904 light mineral oil suitable for PCR (Sigma‒Aldrich, MO, U.S.A.). In the final step, we mixed the washed complex with RT‒qPCR/RT‒LAMP reagents.

#### RT‒qPCR of artificial RNA

We used the Evo M-MLV Reverse Transcription Kit II (with gDNA removal reagent for qPCR) made by Accurate Biotechnology (Changsha, China). We employed both a commercial Applied Biosystems StepOne qPCR instrument (ABI) and a microfluidic chip sample to test the ability of the system to target RNA from the N gene of SARS-CoV-2. An ≈ 100 μl reaction mixture was prepared consisting of 50 μl of 2× RT‒PCR buffer, 4 μl of forward and reverse primers (0.1 mM each) (Table 2), 2 μl of Pro TaqHS polymerase (5 U·µl^−1^), 2 μl of M-MLV reverse transcriptase (5 U·µl^−1^), 4 μl of EvaGreen 20× (25 μM) in deionized H_2_O (DI-H_2_O), 10 μl of the RNA sample, and 24 μl of DI-H_2_O.The RT‒PCR protocol was as follows:

- RT step at 42 °C for 5 min
- Hot start at 95 °C for 30 s
- PCR cycling (40 cycles)
  ○ Denaturation at 95 °C for 15 s
  ○ Annealing and extension at 62 °C for 75 s
- Melting curve analysis (MCA).

After completing the PCR, we conducted an MCA to determine the melting temperature (*T*_M_) of the amplicon, thereby validating the specificity of the PCR amplification. Additionally, we extracted the threshold cycle (*C*_T_) value from the PCR amplification data.

**Table 2:** RT‒PCR primer sequences

Name	Sequence (5’- 3’)
N3-Fw	GGGAGCCTTGAATACACCAAAA
N3-Rv	TGTAGCACGATTGCAGCATTG

#### RT-LAMP reaction with a fluorescence-based readout

We conducted parallel RT-LAMP tests using an RT-LAMP kit from New England BioLabs, Ltd. (Beijing, China), along with six primers targeting the N-A region. These tests were performed on a commercial qPCR instrument maintained at a constant temperature of 65 °C for 60 min, during which we recorded the fluorescence amplitude (*F*) every 30 s. The reaction mixture volume was set at 25 µl, comprising 12.5 µl of RT-LAMP master mix, 0.5 µl of 50× fluorescent dye, 2.5 µl of RT-LAMP primer mix (10×), 4.5 µl of DI-H_2_O, and 5 µl of synthesized target RNA solution. We achieved a range of RNA concentrations by diluting the RNA sample in a 10-fold serial manner, from 10^8^ copies·µl^−1^ to 10^4^ copies·µl^−1^. Each diluted RNA solution was subjected to three separate reaction tests to generate accurate standard curve. We always ran a negative control (NTC) once we had prepared the samples, as we benchmarked them using a commercial StepOne qPCR system made by Applied Biosystems, Inc. (ABI) for both RT‒PCR and RT‒LAMP. Since the NTC results in the ABI qPCR system were negative, we have proven that the samples were not contaminated; thus, we did not analyze the NTC using the chips.

We prepared the RT-LAMP solution for microfluidic chip testing in a manner similar to the previous method, with slight modifications. These included the addition of 5 µl of bovine serum albumin (BSA) solution at a concentration of ≈ 20 mg·ml^−1^ and ≈ 10 µl of synthesized target RNA solution. The RNA concentrations ranged from 10^9^ to 10^5^ copies·µl^−1^ and were adjusted in 100-fold increments. The use of relatively high RNA concentrations aimed to address concerns about exceeding the system limit of quantification (LoQ). This approach ensures that *C*_T_ values derived from high concentrations of DNA or RNA are sufficiently reliable for plotting standard curves ^43^.

The six RT-LAMP primers used were designed using software (https://www.neb.com/neb-primer-de-sign-tools) (Table 3). All primers were synthesized by Sangon Biotech Co., Ltd. (Shanghai, China).

**Table 3:** RT-LAMP primer sequences

Name	Sequence (5’- 3’)
N-A (5’- 3’)	FIP TCTGGCCCAGTTCCTAGGTAGTCCAGACGAATTCGTGGTGG BIP AGACGGCATCATATGGGTTGCACGGGTGCCAATGTGATCT F3 TGGCTACTACCGAAGAGCT B3 TGCAGCATTGTTAGCAGGAT LF GGACTGAGATCTTTCATTTTACCGT LB ACTGAGGGAGCCTTGAATACA

## Results

### Heating/cooling rate

**Fig. 4:**
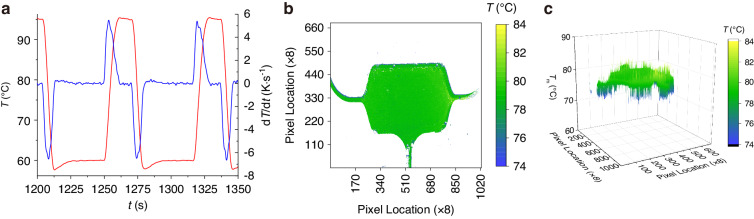
Temperature profiling and distribution in the PCR amplification process. **a** PCR protocol: Graphical representation of the PCR protocol indicating rapid heating (blue line) and cooling (red line) cycles at rates of ≈ 5.5 K·s^−1^ and ≈ −6.7 K·s^−1^, respectively, which are essential for efficient DNA amplification. **b** Infrared radiation intensity: An infrared image capturing the distribution of radiation intensity emitted from the sample area used to infer the temperature distribution across the microfluidic chip. **c** Melting curve analysis (MCA) and temperature mapping: 3D map derived from the MCA showing the extracted apparent melting temperature (*T*_M_*) values across the sample area with a mean ± standard deviation of (79.02 ± 0.77) °C (mean value ± standard deviation), demonstrating uniformity in the thermal profile essential for precise nucleic acid amplification

We initiated testing of the system by optimizing the PID values through an empirical tuning method. The protocol involved two steps: denaturation and annealing, set at 93 °C and 60 °C for 10 s each. Initially, with the integral (*I*) and derivative (*D*) parameters set to zero, we gradually increased the proportional (*P*) gain until the system exhibited instability and oscillation. After observing this difference, we reduced the *P* value and progressively increased the *I* and *D* values. This adjustment continued until the system displayed satisfactory behavior, which we defined by achieving the highest possible heating and cooling rates of 5.5 K·s^−1^ and −6.7 K·s^−1^, respectively, as shown in Fig. 4a.

### Chip temperature uniformity/temperature calibration

The temperature of devices is often determined by noncontact methods such as infrared (IR) imaging via the use of thermal cameras that operate between wavelengths of 8 µm and 14 µm^44^. However, IR systems do not measure the temperature itself but rather the amplitude of emitted radiation influenced by surface properties such as emissivity (ε), in accordance with the Stefan–Boltzmann law. A significant challenge arises even when the exact value of ε is known. This is because the measured radiation comes from the surface of an ≈ 3 mm thick PMMA device, which may have a different temperature than the solution in the chamber where PCR/LAMP processes take place.

A previous proposal introduced a noncontact, photobleaching-free MCA-based measurement method^45^, which has since become routine for measuring temperature inside microfluidic systems, either measuring heat transfer^46^ or calibrating temperature sensors in PCR systems^47^ and microcalorimeters^48^. This MCA-based method, which focuses on the melting curve analysis of double-stranded DNA (dsDNA), provides a single temperature measurement. Its precision remains unaffected by surface properties or photobleaching, unlike commonly used IR^44^ or fluorescein-based methods ^49^.

The tested solution consisted of PCR master mix after completing PCR. This mixture included DNA with a known *T*_M_ value of 79.7 °C, as measured by a commercial qPCR system using EvaGreen intercalating dye (Supplementary Sections D, E). We filled the chip with this solution and placed it in the heated area of the system. The PCR/LAMP chamber within the chip was situated beneath a fluorescent imaging system. This system comprised an LED with a nominal wavelength of 470 nm, an FITC filter set, and a commercial macrolens with a 180 mm focal length set to an aperture of eight. The setup also included a single-lens reflex camera with a 45-megapixel imaging chip. We captured fluorescence images from the chip at temperatures ranging from 70 °C to 84 °C (Supplementary Section F) and processed the data as described previously^47^. The *F* value was measured for random pixels and their adjacent pixels, averaging the *F* values for eight adjacent pixels. We then binned these measurements into 8 × 8 pixels and plotted apparent *T*_M_* values based on location (Fig. 4b). This process resulted in a 3D map (Fig. 4c) showing that the chip’s temperature was uniform at (79.02 ± 0.77) °C (mean ± standard deviation). The minor discrepancy of ≈ 0.68 °C between the mean *T*_M_* values and the *T*_M_ from the commercial PCR machine indicates good agreement between the two temperature sensors. The standard deviation, although small, is thought to be primarily due to image noise rather than actual temperature differences as shown in the MCA image ^47^.

### Sample extraction test on a chip (with external PCR amplification)

We initially conducted tests to verify the nucleic acid extraction efficiency of our chip. Five different DNA concentrations ranging from 297 to 2.97 × 10^6^ copies·µl^−1^ were prepared and divided into two groups, one for the microfluidic chip sample extraction experiment and the other for reference.

**Fig. 5:**
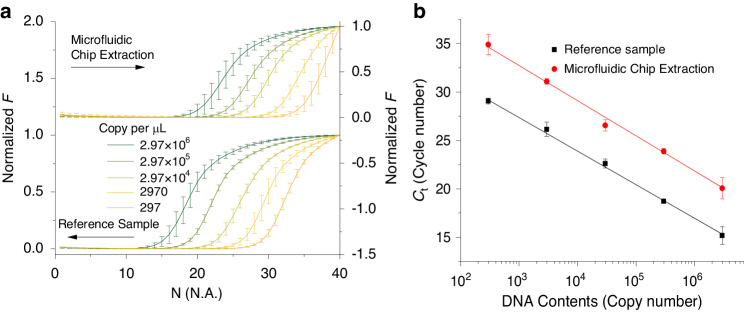
PCR amplification efficiency and standard curve analysis. **a** Amplification curves generated using a commercial qPCR system showing amplification curves of DNA samples after extraction via a microfluidic chip benchmarked against original DNA samples. **b** Standard PCR curves extracted from the PCR amplification curves shown in (**a**) with an extracted Ct shift value of ≈ 4.9, indicating a sample extraction efficiency of ≈ 3.4%

We sequentially introduced the reagents into the respective chambers into the chip 10 μl of oil, 10 μl of elution buffer, 40 μl of a mixture consisting of 10 μl of DNA, 20 μl of magnetic beads as previously described^42^, and 10 μl of lysate at a flow rate of 100 μl·min^−1^. We stored the DNA samples after sample extraction in a 4 °C refrigerator for subsequent external qPCR analysis via commercial qPCR (ABI) according to the following protocol: 0.3 μl of Takara Taq (5 U·μl^−1^), 1 µl of 10 × PCR buffer (Mg^2+^), 0.8 μl of dNTP mixture (2.5 mM), 0.4 μl of forward primer (10 μM), 0.4 μl of reverse primer 10 μM, 0.5 μl of EvaGreen (20×), 1 μl of DNA template, and 5.6 μl of DI-H_2_O. The other group of DNA samples without an in-chip extraction step was prepared with the same protocol and the same instrument (Fig. 5a).

Both standard curves had nearly identical slopes (Fig. 5b), with values of (−3.47 ± 0.08) cycle·dec^−1^ and (−3.63 ± 0.16) cycle·dec^−1^ for PCR of the reference sample solution and PCR of the sample after extraction in the microfluidic chip, respectively. The *C*_T_ value slope obtained from DNA extracted on the chip was greater by ≈ 4.9 cycles in comparison with that of the original samples, indicating ≈ 96.6% DNA loss during extraction. We tested the system with a minimum copy number of 297 copies, as the system was still in its dynamic range even with this sample loss.

### RT‒qPCR results

We prepared an ≈100 µL RT‒PCR master mix with an RNA sample concentration of 1.314 × 10^5^ copies·µL^−1^ following the protocol and temperature guidelines outlined in the previous section. The master mix was divided into two portions. The first portion, consisting of approximately ≈10 µl each, was allocated for three parallel experiments using a commercial qPCR system. The second portion, approximately ≈ 70 µl in volume, was designated for experiments conducted within the microfluidic chips.

**Fig. 6:**
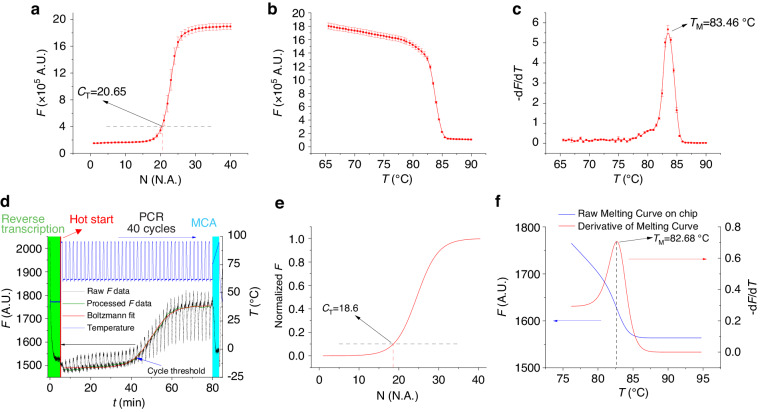
Comparative analysis of RT‒PCR results and melting curves from commercial qPCR machine and microfluidic chip. **a** Commercial qPCR raw data: Amplification plots of N gene RNA sequences performed in triplicate on a standard qPCR machine. **b** MCA on a commercial machine: Post-RT‒PCR MCA showing the specific melting temperatures of the amplified products. **c** Derivative of melting curves: Graphical derivative representation of the melting curves highlighting the peaks corresponding to the specific melting points. **d** Microfluidic chip raw data: Amplification plots from RT‒PCR performed on a microfluidic chip. **e** Normalized chip data: RT‒PCR data from the microfluidic chip after normalization and Boltzmann fitting, revealing the amplification efficiency. **f** Chip MCA results: Melting curve analysis derived from the microfluidic chip’s RT‒PCR raw data indicating the melting temperature of the nucleic acids postamplification, revealing the amplification specificity

Using the commercial qPCR system, we obtained three sets of results. We plotted these results to calculate the mean *F* value along with the corresponding error bar (Fig. 6a). This resulted in a *C*_T_ value of (20.65 ± 0.58) cycles (mean value ± standard deviation). Following RT‒PCR, we conducted an MCA at a temperature ranging from 65 °C to 92 °C. This analysis yielded raw MCA data, from which we derived a *T*_M_ value of (83.46 ± 0.02) °C (mean value ± fitting error) based on the derivative of the curve (Fig. 6b, c).

We subsequently conducted the assay using a microfluidic chip with a chamber volume of ≈6 µl. Therefore, the ≈70 µl volume of the master mix was sufficient for seven experiments. The procedure began by filling almost the entire chip, except for the reaction chamber, with ≈ 40 µl of M5904 mineral oil to prevent evaporation of the RT‒PCR master mix. After introducing ≈ 10 µl of the RT‒PCR master mix into the reaction chamber, we placed the chip into the sample-to-answer system. This system runs a temperature protocol identical to the commercial protocol, employing a continuous method for monitoring both fluorescence and temperature^50^. We recorded temperature and fluorescence data at approximately 0.5 s intervals.

We processed the raw data (Fig. 6d) using our MATLAB code. After normalization and Boltzmann curve fitting (described in the next section), we obtained a *C*_T_ value of (18.6 ± 0.01) cycles and a *T*_M_ value of (82.68 ± 0.05) °C, both (of which are mean values ± fitting errors) (Fig. 6e, f). The results indicated that the RT‒PCR system performed similarly to the commercial qPCR system when the temperature difference was less than 1 °C.

### RT-LAMP results

#### Off-chip RT-LAMP results

**Fig. 7:**
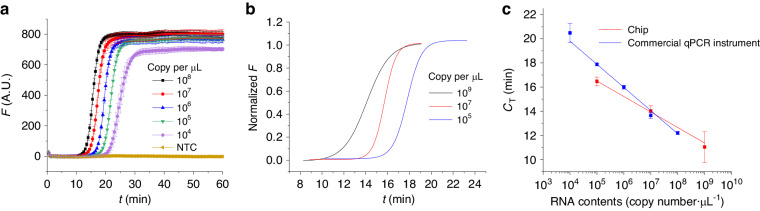
RT-LAMP amplification efficiency and standard curve evaluation. **a** RT-LAMP amplification curves: Real-time amplification data from RT-LAMP reactions performed on a commercial qPCR machine with five different RNA concentrations tested in triplicate, including no-template controls (NTCs). **b** Normalized RT-LAMP data: The raw RT-LAMP amplification data were analyzed on chips and normalized using Boltzmann function curve fitting to demonstrate the amplification progress and efficiency. **c** Standard RT-LAMP curves: Comparative standard curves showing the amplification efficiency of RT-LAMP assays. The left slope represents the standard curve obtained from the commercial qPCR machine with a slope of (–1.913 ± 0.053) min·dec^−1^, and the right slope shows the standard curve derived from the microfluidic chip data with a slope of (–1.282 ± 0.077) min·dec^−1^, both indicating the mean value and fitting error

We conducted RT-LAMP tests with a commercial qPCR system using five different RNA template concentrations, including an NTC. The concentration of each sample was tested in triplicate. For the RT-LAMP process, we maintained the sample at ≈65 °C for 60 min while recording the fluorescence amplitude every ≈30 s. Instead of using cycle numbers, we expressed the collected data as a function of time (*t*). For each RNA concentration, we calculated the mean *F* value along with its standard deviation and plotted these mean values with error bars (Fig. 7a). As previously described, we normalized the data by fitting the mean value of *F* using the Boltzmann function ^51^:

1 $$F={F}_{2}+\frac{{F}_{1}-{F}_{2}}{1+{e}^{\frac{t-{t}_{0}}{{F}_{0}}}}$$

where *F*_1_ is the minimum value of *F*, *F*_2_ is the maximum value of *F*, and *F*_0_ defines the fluorescence slope at inflection point *t*_0_ (Fig. 7b). Subsequently, we subtracted the value of *F*_1_ from the fitted curve by Eq. (1) and then divided it by (*F*_1_–*F*_2_) to normalize it to the minimum value of zero. We defined the *C*_T_ value as the cycle number having a 10% *F* value increase over the baseline. From Eq. (1), we derived *C*_T_ as follows:

2 $${C}_{{\rm{T}}}={t}_{0}-{F}_{0}\cdot \mathrm{ln}\left(\frac{{F}_{1}-{F}_{2}}{F-{F}_{2}}+1\right)$$

by substituting (*F*_1_–*F*_2_)/10 + *F*_1_ for *F*. Then, we plotted (Fig. 7c) the RT-LAMP standard curve (blue) and obtained a slope of (–1.913 ± 0.053) min·dec^−1^ (mean value ± fitting error).

#### RT-LAMP results inside the chip

We initiated the RT-LAMP process by injecting ≈40 µl of M5904 mineral oil into the oil channel through the oil injection port. This step was essential for preventing the RT-LAMP solution from evaporating. Then, we introduced ≈10 µl of the RT-LAMP reaction mixture into the amplification chamber of the chip. The chip was placed in the sample-to-answer system, after which the temperature was maintained at ≈65 °C for a minimum duration of ≈30 min. This process ensured the effective performance of RT-LAMP inside the chip while continuously monitoring the excitation fluorescence signal through the optical components of the system.

The distribution of on-chip RT-LAMP results involved dividing the strains into three groups, each characterized by different RNA concentrations. We ran each sample within the same RNA content group three times, consuming a total of nine chips for these tests. The raw data of these tests are presented in supplementary Section G. Individual normalization of the captured curves from the raw data of the chip amplification was performed (Fig. 7b), leading to the creation of the RT-LAMP standard curve (red curve) (Fig. 7c). This curve displayed a slope of (–1.282 ± 0.077) min·dec^−1^ (mean value ± fitting error), similar to the benchmark data obtained from the commercial qPCR system, which had a slope of (–1.913 ± 0.053) min·dec^−1^ (mean value ± fitting error). The square of the Pearson correlation coefficient for the fitting curve in the chip was 0.996, whereas it was 0.998 in the commercial qPCR system, indicating good standard curve linearity. These results demonstrated the effective performance of RT-LAMP on the chip, as evidenced by the establishment of the RT-LAMP standard curve.

#### Integrated system

The operation of the integrated system involved a sequence of steps. We began by introducing ≈1 µl of magnetic bead solution into ≈10 µL of RNA solution. Uniform mixing was achieved by pipetting the mixture for ≈30 s. This prepared solution was then injected into the sample-injection aperture. Pumping continued until the mixture reached the periphery of the oil chamber.

In the next phase, we pipetted ≈10 µL of the RT-LAMP master mix into the chip’s reaction chamber via the sample input port to fill the chamber. Subsequently, we pipetted ≈2 µl of M5904 mineral oil to fill the oil chamber and the channel. After completing these sample injection stages, we positioned the chip on the sample-to-answer system with the arm having a permanent magnet above the sample extraction chamber. Subsequently, we pipetted the sample to a volume of ≈5 µl in ≈5 min, after which all the other fluid inputs/outputs were blocked except for that for the raw sample waste (Fig. 3b). The magnet captured the particles with bound RNA and moved via the oil chamber, effectively removing all the debris from the solution, leaving only magnetic particles with bound RNA. Then, we moved the magnetic beads into the reaction chamber containing RT-LAMP master mix and initiated the RT-LAMP protocol to facilitate the amplification procedure. This entire sample pretreatment required ≈8 min, including ≈5 min for sample loading and ≈3 min for nucleic acid extraction.

**Fig. 8:**
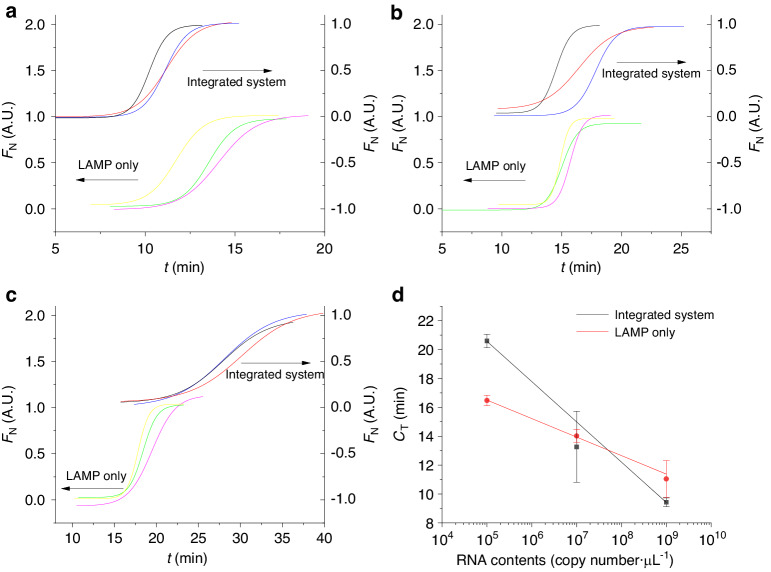
Standard curve analysis of the integrated RT-LAMP system. Normalized Boltzmann function curve derived from raw RT-LAMP data from the chip without sample extraction and with the function of the integrated system with RNA contents of **a** 10^9^ copies·µl^−1^, **b** 10^7^ copies·µl^−1^, and **c** 10^5^ copies·µl^−1^; each experiment was conducted in triplicate, and the results are shown as different color curves. **d** The standard curve (black) shows a slope of (–2.788 ± 0.098) min·dec^−1^ (mean value ± fitting error), which is indicative of the system’s amplification capability after sample extraction. This curve is compared to the red curve with a slope of (–1.282 ± 0.077) min·dec^−1^ from the chip without the sample extraction process, emphasizing the influence of the integrated extraction on the amplification efficiency

We tested RNA sample concentrations of 10^5^, 10^7^, and 10^9^ copies·µl^−1^, and each concentration was tested in triplicate to establish the standard curve of the integrated system (Fig. 8). The chips were disposable, and nine chips were used for nine experiments. We extracted *C*_T_ values via the same procedure as mentioned in the previous section, and the integrated system demonstrated a LAMP amplification slope of (–2.788 ± 0.098) min·dec^−1^ (mean value ± fitting error), as illustrated in Fig. 8d. The fitting error of the curve was acceptable even though the middle point (concentration of 10^7^ copies·µl^−1^) was off the fitting point, but the curve itself contained a test error bar. These results indicate that the system is capable of performing RT-LAMP following the extraction function of the integrated system and enables relative quantification with a standard curve comparable to that of a commercial qPCR system.

## Discussion

Our study introduces a rapid and versatile sample-to-answer system designed for automated nucleic acid extraction and analysis. This platform uses both RT‒qPCR and RT‒LAMP methods and offers a simplified workflow suitable for real-time molecular diagnostics ^52^.

An important feature of this system is the controlled heating and cooling rate^53^. This aspect is crucial for PCR-based techniques that depend on specific temperatures for enzymatic reactions^54^. In this study, we optimized only the PID constants to achieve the fastest possible heating and cooling rates that the system hardware allows, at 5.5 K·s^−1^ and −6.7 K·s^−1^, respectively. However, this system optimization was not accompanied by PCR protocol optimization. We employed a conservative PCR protocol with rather long denaturation and annealing/extension steps to demonstrate that the system can also be applied to perform RT‒PCR analysis, the gold standard in DNA/RNA diagnostics. Modifications to the microfluidic chip to improve heat transfer are essential for running a reasonably fast RT‒PCR protocol.

Precise temperature management during PCR-based amplification can significantly influence diagnostic sensitivity and specificity^55^. Here, we implemented a noncontact, photobleaching-free MCA-based measurement technique^45,47,48^. This technique excels at providing accurate and consistent temperature readings^56^ and is a significant advantage over traditional methods that often encounter issues related to surface properties and potential photobleaching effects.

A critical step in molecular diagnostics is nucleic acid extraction^57^. Our system performs on-chip extraction, but some loss of RNA was observed, indicating the need for further optimization. The extraction process is reasonably good at detecting the presence of the target nucleic acids even at lower concentration sensitivities. We tested the system with a minimum copy number of 297 copies, as the system was still in its dynamic range even with this sample loss. This limit is sufficient for SARS-CoV-2 screening, as the method was conducted in China^58^ in 2023 even when the pooling technique was used^59^ to further dilute the sample by combining 20 samples from different individuals. This indicates that in such a case, our system limit of extraction being better than 297 would be sufficient for detecting the presence of SARS-CoV-2 RNA^60^. However, additional diluted samples should be tested because certain viruses, such as human immunodeficiency virus, have a low viral load, and monitoring the number of RNA copies indicates HIV treatment efficacy ^61^.

The results from our on-chip system closely align with those from commercial RT‒qPCR and RT‒LAMP platforms, confirming the robustness of our approach. The on-chip RT-LAMP method, known for its speed and high amplification efficiency, shows strong potential for field applications ^36,62^.

## Conclusion

Our study introduces a rapid, versatile, and low-cost sample-to-answer system for SARS-CoV-2 diagnostics. It integrates the extraction and purification of nucleic acids, followed by amplification using either RT‒qPCR or RT‒LAMP. This versatility, coupled with the use of conventional chemistry, makes the system competitive with most FDA-approved devices, completing analyses within 28 min. This timeframe includes ≈5 min for sample loading, ≈3 min for target extraction and ≈20 min for RT-LAMP. However, the RT‒PCR protocol is significantly slower because the heat transfer of the microfluidic chip is not optimized. We demonstrated the RT-PCR capability of the chip, but it is not the system’s primary focus. The total consumption of either the RT-LAMP or RT‒PCR master mix was only ≈10 µl. Currently, the major factor influencing detection limits is a high level of NA loss during the extraction step, which was calculated to be 96.6%. Even with such low extraction efficiency, the system is still sufficiently sensitive for COVID-19 diagnosis due to the high viral load of SARS-CoV-2 but is not adequate for identifying HIV and similar viruses with low viral loads.

The PMMA-based sample-to-answer chip developed in this work enables mass production without complex treatment procedures, leading to a lower cost of ≈$9.5 per test compared to commercial options. This cost can be further reduced by replacing CNC-based chip manufacturing techniques with more economical methods, such as roll-to-roll hot embossing or injection molding. This system requires further refinement, especially for minimizing RNA loss, and it has robust and high performance. Its portability, ease of use, and automation capabilities make the platform suitable for on-site diagnosis by minimally trained personnel, eliminating the need for centralized facilities. This study lays the groundwork for the development of more sophisticated, reliable, and user-friendly microfluidic systems for molecular diagnostic testing.

### Supplementary information

Technical Details

## References

[1] Huang C (2020). Clinical features of patients infected with 2019 novel coronavirus in Wuhan, China. Lancet.

[2] Zhu H, Fohlerová Z, Pekárek J, Basova E, Neužil P (2020). Recent advances in lab-on-a-chip technologies for viral diagnosis. Biosens. Bioelectron..

[3] Kilic A, Hiestand B, Palavecino E (2021). Evaluation of performance of the BD veritor SARS-CoV-2 chromatographic immunoassay test in patients with symptoms of COVID-19. J. Clin. Microbiol..

[4] Kang T, Lu J, Yu T, Long Y, Liu G (2022). Advances in nucleic acid amplification techniques (NAATs): COVID-19 point-of-care diagnostics as an example. Biosens. Bioelectron..

[5] Wu L (2020). Fluidic multivalent membrane nanointerface enables synergetic enrichment of circulating tumor cells with high efficiency and viability. J. Am. Chem. Soc..

[6] Zhang H, Pajer P, Kudr J, Zitka O, Neužil P (2021). Design considerations for point-of-need devices based on nucleic acid amplification for COVID-19 diagnostics and beyond. Biotechniques.

[7] Heger LA (2022). Clinical analysis on diagnostic accuracy of Bosch Vivalytic SARS-CoV-2 point-of-care test and evaluation of cycle threshold at admission for COVID-19 risk assessment. BMC Infect. Dis..

[8] Hogan CA (2020). Comparison of the Accula SARS-CoV-2 test with a laboratory-developed assay for detection of SARS-CoV-2 RNA in clinical nasopharyngeal specimens. J. Clin. Microbiol..

[9] Hansen G (2021). Clinical performance of the point-of-care cobas Liat for detection of SARS-CoV-2 in 20 min: a multicenter study. J. Clin. Microbiol..

[10] Schuler F (2016). Digital droplet PCR on disk. Lab Chip.

[11] Pan Y (2020). Droplet digital PCR enabled by microfluidic impact printing for absolute gene quantification. Talanta.

[12] Zhou SF (2019). A highly integrated real-time digital PCR device for accurate DNA quantitative analysis. Biosens. Bioelectron..

[13] Convergys. *The Convergys® POC RT-PCR COVID-19 Testing Platform (2023 October 13)*, (online) https://convergent-technologies.de/ (2023).

[14] Wu QQ (2011). Integrated glass microdevice for nucleic acid purification, loop-mediated isothermal amplification, and online detection. Anal. Chem..

[15] Zhang C, Xing D, Li Y-Y (2007). Micropumps, microvalves, and micromixers within PCR microfluidic chips: advances and trends. Biotechnol. Adv..

[16] Kim D, Rho HS, Jambovane S, Shin S, Hong J (2016). Evaluation of peristaltic micromixers for highly integrated microfluidic systems. Rev. Sci. Instrum..

[17] Soares R (2021). Sample-to-answer COVID-19 nucleic acid testing using a low-cost centrifugal microfluidic platform with bead-based signal enhancement and smartphone read-out. Lab Chip.

[18] Stumpf F (2016). LabDisk with complete reagent prestorage for sample-to-answer nucleic acid based detection of respiratory pathogens verified with influenza A H3N2 virus. Lab Chip.

[19] Zhang L (2018). Hand-powered centrifugal microfluidic platform inspired by the spinning top for sample-to-answer diagnostics of nucleic acids. Lab Chip.

[20] Li L, Miao B, Li Z, Sun Z, Peng N (2019). Sample-to-Answer HBV DNA detection from whole blood on a centrifugal microfluidic platform with double rotation axes. ACS Sens..

[21] Loo J (2017). Sample-to-answer on molecular diagnosis of bacterial infection using integrated lab-on—a-disc. Biosens. Bioelectron..

[22] Tian F (2020). A fully automated centrifugal microfluidic system for sample-to-answer viral nucleic acid testing. Sci. China Chem..

[23] Ji M (2020). Automated multiplex nucleic acid tests for rapid detection of SARS-CoV-2, influenza A and B infection with direct reverse-transcription quantitative PCR (dirRT-qPCR) assay in a centrifugal microfluidic platform. RSC Adv..

[24] Choi J (2015). Paper-based sample-to-answer molecular diagnostic platform for point-of-care diagnostics. Biosens. Bioelectron..

[25] Gan W (2017). Chitosan-modified filter paper for nucleic acid extraction and “in Situ PCR” on a thermoplastic microchip. Anal. Chem..

[26] Chen P (2019). Fully integrated nucleic acid pretreatment, amplification, and detection on a paper chip for identifying EGFR mutations in lung cancer cells. Sens. Actuators B Chem..

[27] Strauss C, Endimiani A, Perreten V (2015). A novel universal DNA labeling and amplification system for rapid microarray-based detection of 117 antibiotic resistance genes in Gram-positive bacteria. J. Microbiol. Methods.

[28] Shlyapnikov YM, Malakhova E, Shlyapnikova E (2019). Rapid amplification-free microarray-based ultrasensitive detection of DNA. Anal. Chem..

[29] Shen K-M (2019). An integrated microfluidic system for rapid detection and multiple subtyping of influenza A viruses by using glycan-coated magnetic beads and RT-PCR. Lab Chip.

[30] Gwida M (2020). Microarray-based detection of resistance and virulence factors in commensal Escherichia coli from livestock and farmers in Egypt. Vet. Microbiol..

[31] Prasad A, Hasan SMA, Grouchy S, Gartia M (2018). DNA microarray analysis using a smartphone to detect the BRCA-1 gene. Analyst.

[32] Jonguitud-Borrego N (2022). High—throughput and automated screening for COVID-19. Front. Med. Technol..

[33] Koo B (2023). Automated sample-to-answer system for rapid and accurate diagnosis of emerging infectious diseases. Sens. Actuators B Chem.

[34] Lu W (2016). High-throughput sample-to-answer detection of DNA/RNA in crude samples within functionalized micro-pipette tips. Biosens. Bioelectron.

[35] Mori Y, Nagamine K, Tomita N, Notomi T (2001). Detection of loop-mediated isothermal amplification reaction by turbidity derived from magnesium pyrophosphate formation. Biochem. Biophys. Res. Commun..

[36] Goto M (2009). Colorimetric detection of loop-mediated isothermal amplification reaction by using hydroxy naphthol blue. Biotechniques.

[37] Velten T (2011). Roll-to-roll hot embossing of microstructures. Microsyst. Technolog..

[38] Becker H, Gärtner C (2008). Polymer microfabrication technologies for microfluidic systems. Anal. Bioanaly. Chem..

[39] Balram KC (2016). The nanolithography toolbox. J. Res. Natl. Inst..

[40] Zhang H (2020). Nanolithography toolbox—Simplifying the design complexity of microfluidic chips. J. Vac. Sci. Technol. B.

[41] Hsu Y-C, Chen T-Y (2007). Applying Taguchi methods for solvent-assisted PMMA bonding technique for static and dynamic μ-TAS devices. Biomed. Microdevices.

[42] Juang DS (2021). Oil immersed lossless total analysis system for integrated RNA extraction and detection of SARS-CoV-2. Nat. Commun..

[43] Herder, J. et al. *Environmental DNA - a review of the possible applications for the detection of (invasive) species*. 10.13140/RG.2.1.4002.1208 (2014).

[44] Roper MG, Easley CJ, Legendre LA, Humphrey JA, Landers JP (2007). Infrared temperature control system for a completely noncontact polymerase chain reaction in microfluidic chips. Anal. Chem..

[45] Neuzil P, Cheng F, Soon JBW, Qian LL, Reboud J (2010). Non-contact fluorescent bleaching-independent method for temperature measurement in microfluidic systems based on DNA melting curves. Lab Chip.

[46] Fohlerova Z (2020). Rapid characterization of biomolecules’ thermal stability in a segmented flow-through optofluidic microsystem. Sci. Rep..

[47] Gaňová M (2022). Temperature non-uniformity detection on dPCR chips and temperature sensor calibration. RSC Adv..

[48] Ni S, Bu Y, Zhu H, Neuzil P, Yobas L (2021). A Sub-nL chip calorimeter and its application to the measurement of the photothermal transduction efficiency of plasmonic nanoparticles. J. Microelectromechanical Syst..

[49] Ross D, Gaitan M, Locascio LE (2001). Temperature measurement in microfluidic systems using a temperature-dependent fluorescent dye. Anal. Chem..

[50] Zhang H (2019). Revealing the secrets of PCR. Sensors Actuators B: Chem..

[51] Zhang H (2021). Determination of advantages and limitations of qPCR duplexing in a single fluorescent channel. ACS Omega.

[52] Yeh E-C (2017). Self-powered integrated microfluidic point-of-care low-cost enabling (SIMPLE) chip. Sci. Adv..

[53] Holland PM, Abramson RD, Watson R, Gelfand DH (1991). Detection of specific polymerase chain reaction product by utilizing the 5’–3’exonuclease activity of Thermus aquaticus DNA polymerase. Proc. Natl Acad. Sci..

[54] Saiki RK (1988). Primer-directed enzymatic amplification of DNA with a thermostable DNA polymerase. Science.

[55] Mackay IM, Arden KE, Nitsche A, Real-time PCR (2002). Real time PCR in virology. Nucleic Acids Res..

[56] Ririe KM, Rasmussen RP, Wittwer CT (1997). Product differentiation by analysis of DNA melting curves during the polymerase chain reaction. Anal. Biochem..

[57] Qiu G (2020). Dual-functional plasmonic photothermal biosensors for highly accurate severe acute respiratory syndrome coronavirus 2 detection. ACS Nano.

[58] Han X (2022). SARS-CoV-2 nucleic acid testing is China’s key pillar of COVID-19 containment. The Lancet.

[59] Deckert A, Bärnighausen T, Kyei NN (2020). Simulation of pooled-sample analysis strategies for COVID-19 mass testing. Bull. World Health Org..

[60] Zhou D, Zhou M (2022). Mathematical model and optimization methods of wide-scale pooled sample testing for COVID-19. Mathematics.

[61] Estill J (2012). Viral load monitoring of antiretroviral therapy, cohort viral load and HIV transmission in Southern Africa: a mathematical modelling analysis. AIDS.

[62] Nagamine K, Hase T, Notomi T (2002). Accelerated reaction by loop-mediated isothermal amplification using loop primers. Mol. Cell. Probes.

[63] FDA, U. S. F. A. D. A. *Potential for False Results with Roche Molecular Systems, Inc. cobas SARS-CoV-2 & Influenza Test for use on cobas Liat System-Letter to Clinical Laboratory Staff, Point-of-Care Facility Staff, and Health Care Providers*, https://www.fda.gov/medical-devices/letters-health-care-providers/potential-false-results-roche-molecular-systems-inc-cobas-sars-cov-2-influenza-test-use-cobas-liat (2021).

