# Supplementary material for: An integrated microfluidic platform for nucleic acid testing
Source: Microsyst Nanoeng. 2024 May 23;10:66. doi: 10.1038/s41378-024-00677-6 (PMC11111744; doi:10.1038/s41378-024-00677-6)
Supplement: Supplementary file 1 — Technical Details [file 41378_2024_677_MOESM1_ESM.docx]

# A microfluidic based sample-to-answer system for nucleic acid extraction and detection

## Authors

Antao Sun^1*^, Petra Vopařilová^2*^, Xiaocheng Liu^1^, Bingqian Kou^1^, Tomáš Řezníček^3^, Tomáš Lednický^4^, Sheng Ni^5^, Jiří Kudr^2^, Ondřej Zítka^2^, Zdenka Fohlerová^6^, Petr Pajer^7^, Haoqing Zhang^8.9#^ and Pavel Neužil^1#^

## Affiliations

^1^Ministry of Education Key Laboratory of Micro and Nano Systems for Aerospace; School of Mechanical Engineering, Northwestern Polytechnical University; 127 West Youyi Road, Xi’an, Shaanxi 710072, P.R. China

^2^Department of Chemistry and Biochemistry, Mendel University in Brno, Zemědělská 1, 61300, Brno, Czech Republic

^3^ITD Tech s.r.o, Osvoboditelu 1005, 735 81 Bohumín, Czech Republic

^4^Central European Institute of Technology, Brno University of Technology, Purkyňova 123, Brno 61200, Czech Republic

^5^Department of Electronic and Computer Engineering, The Hong Kong University of Science and Technology, Clear Water Bay, Kowloon, Hong Kong SAR, China

^6^Department of Microelectronics, Faculty of Electrical Engineering and Communication, Brno University of Technology, Technická 3058/10, Brno, 61600, Czech Republic

^7^Military Health Institute, U Vojenské nemocnice 1200, 16200, Praha 6, Czech Republic

^8^The Key Laboratory of Biomedical Information Engineering of Ministry of Education; School of Life Science and Technology, Xi’an Jiaotong University; Xi’an, Shaanxi 710049, P.R. China

^9^Bioinspired Engineering and Biomechanics Center (BEBC), Xi’an Jiaotong University, Xi'an 710049, P.R. China

^*^These two co-authors contributed equally and thus they are both considered as first authors.

Corresponding author: [pavel.neuzil@nwpu.edu.cn](mailto:pavel.neuzil@nwpu.edu.cn), [zhanghaoqing@xjtu.edu.cn](mailto:zhanghaoqing@xjtu.edu.cn)

## Supplementary Section A: sample-to-answer system setup

*Figure S 1 (A) Photograph of the complete system with the microfluidic chip. (B) Block diagram with all the essential electrical components of the system.*

## Supplementary Section B: sample-to-answer chip description


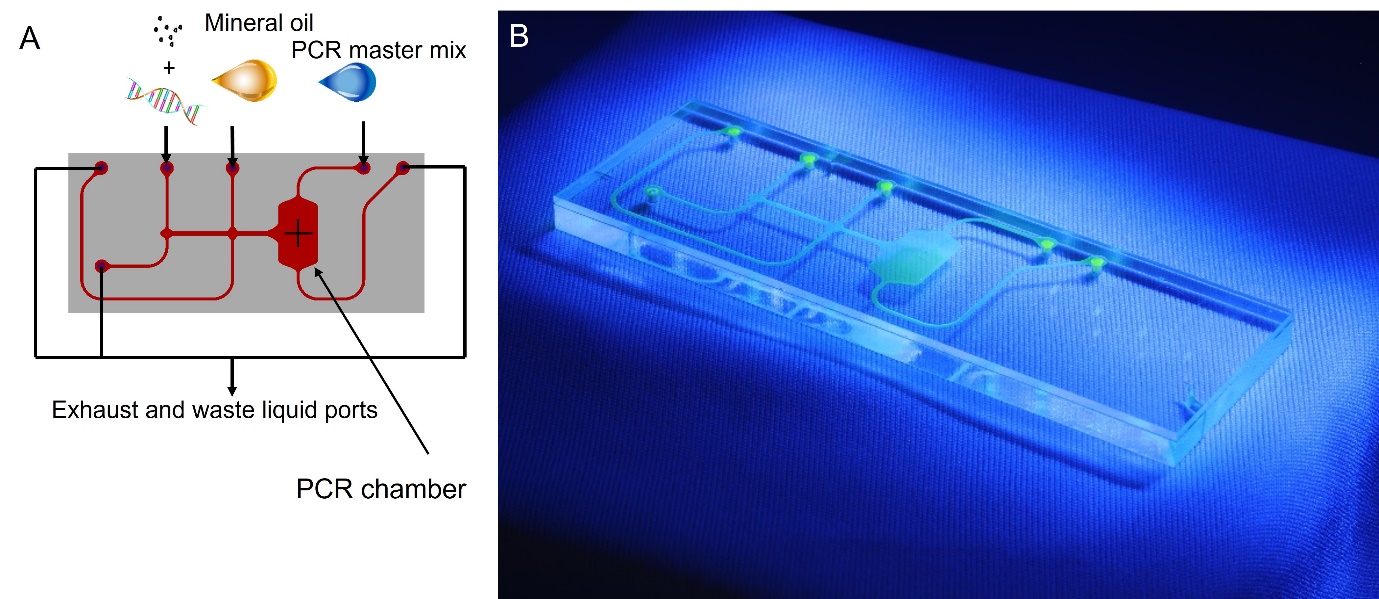


*Figure S 2 Nucleic acid extraction chip. (A) The chip layout with the principle of its operation shows a sample, oil, polymerase chain reaction (PCR) master mix inputs, and PCR chamber. (B) Poly(methylmethacrylate) chip designed using the computed numerical control technique capped by chemically assisted thermal bonding.*

## Supplementary Section C: optical detection module


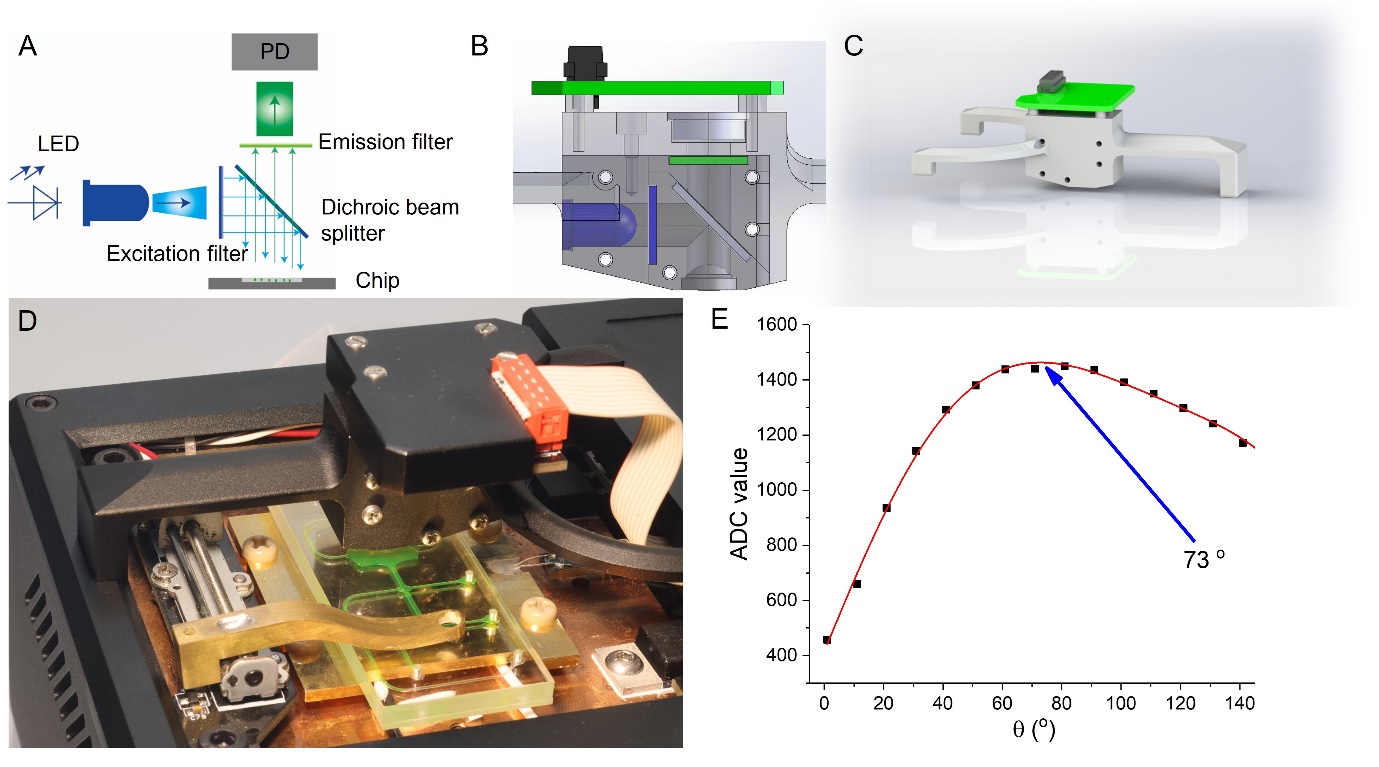


*Figure S 3 System optics for fluorescence detection. (A) Schematic diagram of the system consisting of an LED with an excitation filter, dichroic beam splitter interacting with a microfluidic chip, and the photodiode monitoring excited fluorescence via an emission filter. (B) A cross-section of the optical system (C) Computer-aided design drawing of the system with a printed circuit board (green) containing essential processing of a photocurrent from the photodiode. (D) Photograph of a fabricated and assembled optical system mounted on a sample-to-answer device. (E) The optimum phase shift of the lock-in amplifier obtaining maximum ADC value found to be 73°.*

## Supplementary Section D: sample-to-answer system software control

*Figure S 4 Software control windows displaying the main functions. (A) Manual control (B) Protocol control (C) Fluorescent measurement setting and data recording (D) Temperature monitoring (E) Stepper motor control*

## Supplementary Section E: MCA for temperature calibration in commercial qPCR instrument

*Table S 1 Protocol for melting curve analysis in ABI*

| **Component** | **Volume** |
| --- | --- |
| *TaKaRa* Taq (5 U·μL^-1^) | 1 µL |
| EvaGreen (20×) | 0.5 µL |
| 10× PCR Buffer (Mg^2+^) | 1 µL |
| dNTP Mixture (2.5 mM each) | 1.5 µL |
| Forward primer (10 μM) | 0.4 µL |
| Reverse primer (10 μM) | 0.4 µL |
| BSA (20 mg·mL ^-1^) | 1 µL |
| DNA | 1 µL |
| dH_2_O | 3.2 µL |
| Total | 10 µL |

*Table S 2 Primers and target DNA sequences*

| Name | Sequence (5’ to 3’) |
| --- | --- |
| E_gene | CCGACGACGACTACTAGCGTGCCTTTGTAAGCACAAGAAAGTGAGTACGAACTTATGTACTCATTCGTTTCGGAAGAAACAGGTACGTTAATAGTTAATAGCGTACTTCTTTTTCTTGCTTTCGTGGTATTCTTGCTAGTCACACTAGCCATCCTTACTGCGCTTCGATTGTGTGCGTACTGCTGCAATATTGTTAACGTGAGTTTAGTAAAACCAACGGTTTACGTCTACTCGCGTGTTAAATTCCTAGCCTGGATTATGTTAC |
| Forward primer | ACAGGTACGTTAATAGTTAATAGCGT |
| Reverse primer | ATATTGCAGCAGTACGCACACA |

*Table S 3 Temperature protocol in ABI*

|  | Hot start | Cycles (cycle numbers: 40) | | Melting curve analysis | |
| --- | --- | --- | --- | --- | --- |
|  |  | Denaturation | Annealing & Extension |  |  |
| Temperature | 95 ℃ | 95 ℃ | 60 ℃ | 65 ℃ | 90 ℃ (+0.5 ℃/s) |
| Time | 10 s | 10 s | 75 s | 10s | / |

**
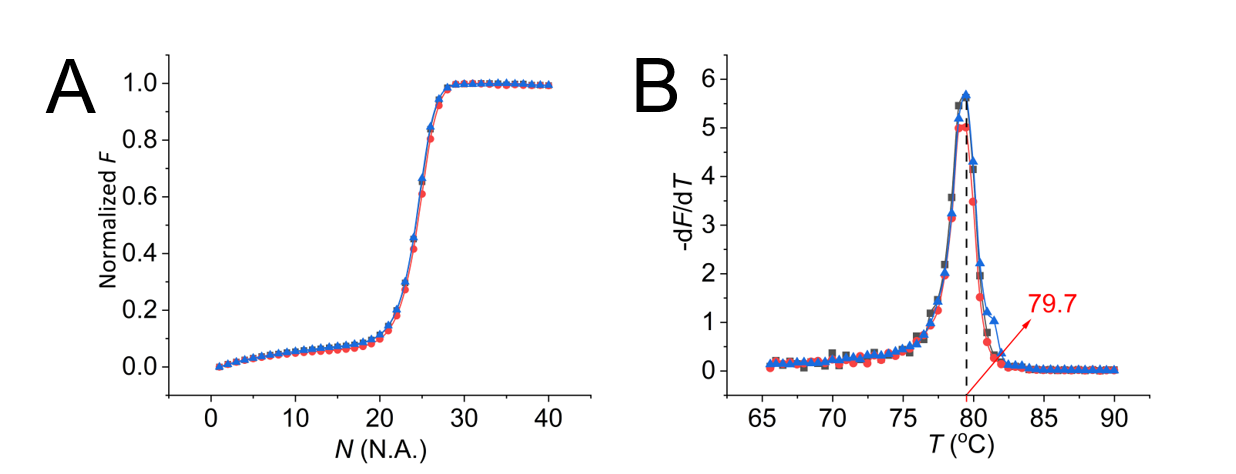
**

*Figure S 5 (A) PCR amplification curve before MCA in commercial qPCR instrument. (B) Derivative MCA curve in commercial qPCR instrument.*

We performed MCA in commercial qPCR instrument obtaining *T*_M_ of (79.70 ± 0.15) °C (mean ± standard deviation).

## Supplementary Section F: MCA on the chip capturing fluorescent images


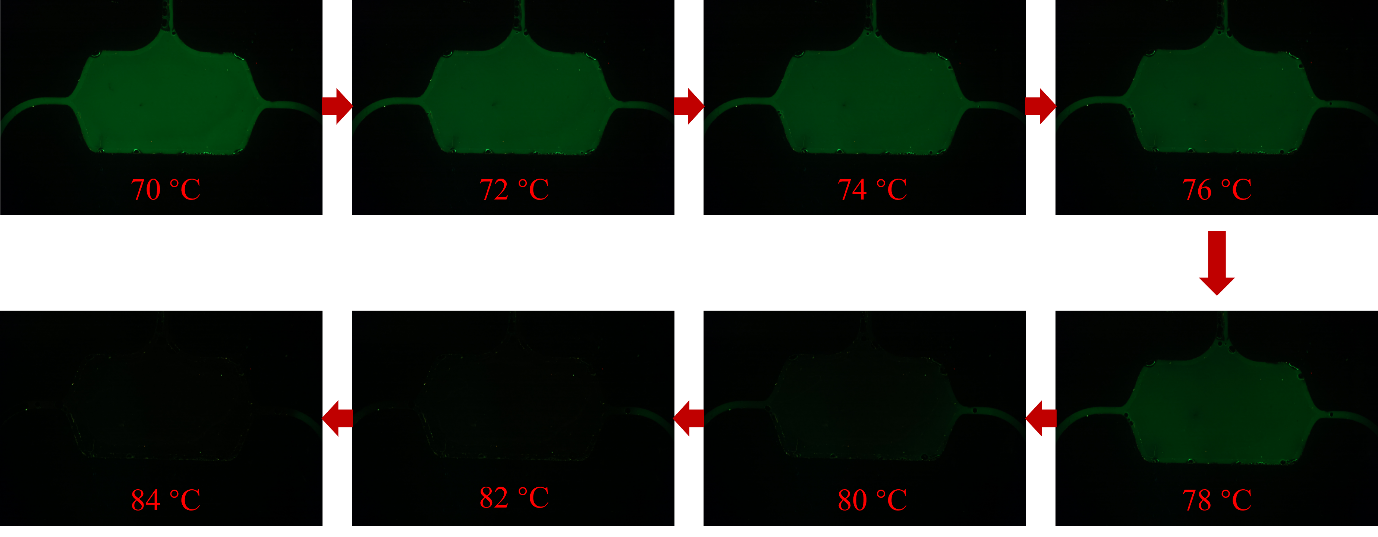


*Figure S 6 Captured fluorescent images from the chip filled with after-PCR master mix at temperatures from* *70 °C to 84 °C in sequence.*

We performed MCA in a chip filled with an after-PCR master mix of known *T*_M_ we measured before using a commercial qPCR instrument. Subsequently, we captured fluorescent images at temperatures from 70°C to 84°C in every 2°C temperature increment and processed the image as shown earlier using Matlab script. After binding 4×4 pixels inside the chamber we extracted the MCA for each cluster of 16 pixels determined the apparent melting temperature and then formed a map of those temperatures showing temperature nonuniformity and temperature sensor offset. This is in the main text of the manuscript.

## Supplementary Section G: MCA on the chip capturing fluorescent images


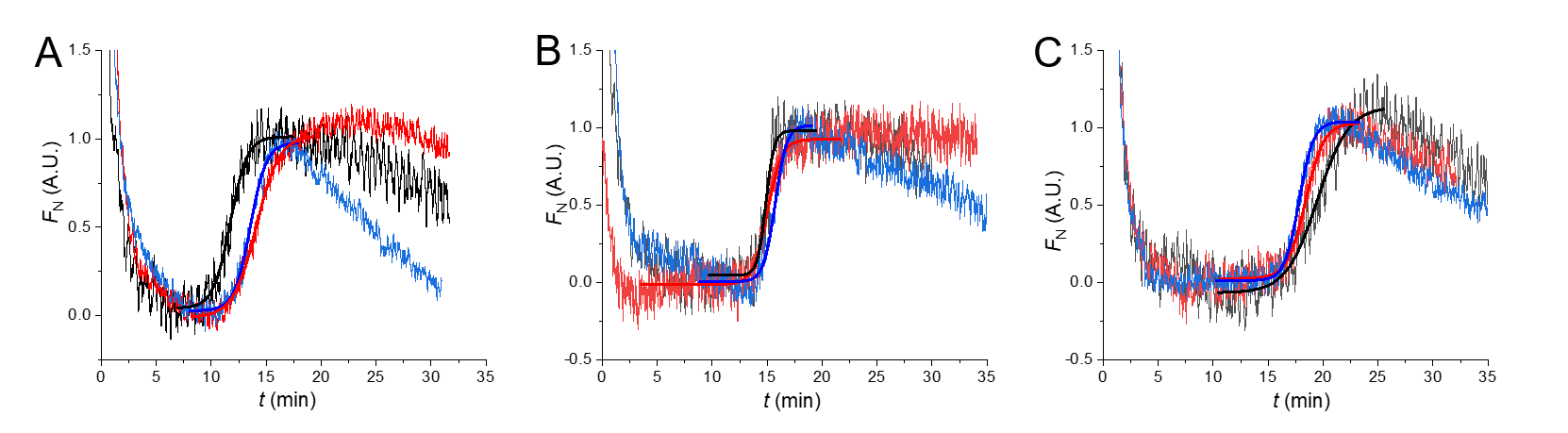


*Figure S 7 (A) (B) (C) Raw data using RT-LAMP in a microfluidic chip with RNA contents of 109 copies·µL^-1^, 107 copies·µL^-1^, and 105 copies·µL^-1^,* *respectively.*
